# Intelligence, Correspondence, &c.

**Published:** 1833-01-01

**Authors:** 


### INTELLIGENCE, CORRESPONDENCE, &c.

We understand that the Central Board of Health is to be immediately dissolved—a fact which gives us much pleasure, not from any hostility to its members, or jealousy of their emoluments or good quarters in Whitehall; but on account of the cause of their medico-political dissolution—the cessation of cholera in the Metropolis, as well as in most other parts of the Kingdom.

The last measure that emanated from the Central Board, was the wisest and the best that was ever engendered there—a recommendation to the Hospitals of the Metropolis, and, consequently, to all other public Institutions of this kind, *to receive cholera patients, in future, as they would patients labouring under any other disease!* This speaks for itself; and happy would it have been for society, in this country, had "CHOLERA HOSPITALS" never been projected, and had the sick been received into the public institutions already in existence. When they were filled, it would have been time enough to construct others as appendages, but not as *pest-houses*, which frightened and prejudiced the poor against such asyla. One thing is clear—that if it be right, in future, to receive cholera patients in public hospitals, it would have been right in times past.

### MEDICO-CHIRURGICAL REVIEW.

Numbers XXVII. and XXXI. of the present Series having been out of print for some time, so that complete sets cannot be made up, the Editor will feel particularly obliged to any Gentleman holding either of these Numbers, and not having the Series, to favour the Publisher (Highley) with the said Numbers, when the full price will be returned, and much obligation conferred.

Communications have been received from Dr. MACROBIN, of Aberdeen—from P. M. LYONS, Esq. of Brighton—from Mr. BULLAR, and others, which are under consideration.

### *Literary Intelligence.*

Dr. BOOTT is preparing for publication, in two octavo volumes, to be published in January, a Memoir of the Life and Medical Opinions of Dr. Armstrong, late Physician of the Fever Institution of London, and author of Practical Illustrations of Typhus and Scarlet Fever; to which will be added, an Inquiry into the Facts connected with those Forms of Fever attributed to Malaria and Marsh Effluvium.

Mr. CURTIS, Aurist to His Majesty, has in the press, besides a new Edition of his Essay on the Deaf and Dumb, a Treatise on the Diseases of the Eye, with new Modes of Treatment; with a Plate, shewing the connexion of the Organs of Hearing and Sight.

